# Supplementary material for: Study on the Measurement and Influencing Factors of Care Service Demand of Disabled Elderly in Urban and Rural China
Source: Int J Environ Res Public Health. 2022 Sep 5;19(17):11112. doi: 10.3390/ijerph191711112 (PMC9518346; doi:10.3390/ijerph191711112)
Supplement: Supplementary file 1 [file ijerph-19-11112-s001.zip › ijerph-1833328-supplementary.pdf]

Supplementary Materials

Table S1. Description of variables in the Measurement And Influencing Factors of Care Service Demand of Disabled Elderly in Urban And Rural China.

Variable			Description	N	Min	Max	Mean	Sd
Explanatory Variable	Tendency Factors	Gender	female=0；male=1	2917	0	1	54.1%	0.498
		Age	Age=2018-the year of birth	2917	60	99	69.54	6.781
		Education	Unschooled=1；Old-style Private School or Primary School=2；Middle School=3；High School or Technical Secondary School=4；College or University Degree=5；Master Degree or Above=6	2916	1	5	2.113	0.939
		Attributes	rural=0；urban=1	2917	0	1	57.4%	0.495
		Worried Pension	The questionnaire asked "what aspects of pension do you worry about?", Select any one of the answers "Daily Care" "Disease Care" or "Spiritual Comfort", which means that you are worried about it and the value is 1, else=0	2917	0	1	48.5%	0.500
	Resource Factors	Economy	The questionnaire asked "how was your family's economic situation in the past year?", Represents the economic situation of the elderly. Very Difficult = 1; Relatively Difficult = 2; Roughly Enough, No Lack = 3; Comparative Margin = 4; Fairly Abundant = 5	2917	1	5	1.968	0.838
		Family Help	The questionnaire asked "how many relatives can you pour out your thoughts and ask for help?",Represents the level of support that can get help from loved ones. None = 1; 1=1; 2==2; 3~4=3; 5~8=4; 9 and above=5	2916	0	5	1.827	1.491
		LCI	The questionnaire asked "if you have long-term care insurance, you can reimburse part of your living care and medical care expenses after participating, but you need to pay a certain amount of insurance premium. Are you willing to participate?", Unwilling to participate = 0, willing to participate = 1	2367	0	1	60.0%	0.490
	Need Factors	Health	The questionnaire asks "how do you feel about your health?", Reverse assignment according to the answer. Very Poor = 1; Comparison Difference = 2; Better = 3; General = 4; Very Good = 5	2675	1	5	2.253	0.934
		Chronic Disease	The questionnaire asks "do you have the following chronic diseases?", Sum up the number of chronic diseases according to the answers. The value is 0 ~ 12, which represents the number of chronic diseases. 0 means "no chronic diseases" , 12 means "12 kinds of chronic diseases"	2917	0	11	3.074	1.727

		Disability Degree	According to the answer of the activity of daily living scale (ADL), if 1 ~ 2 daily activities are difficult or unable to do, it is a mild disability and the value is 1; If 3 ~ 4 daily activities are difficult or unable to do, it is moderately disabled and the value is 2; If 5 ~ 6 daily activities are difficult or unable to do, it is severe disability and the value is 3	2916	1	3	1.693	0.789
		Daily Care	Select the answers of the three types of service items in the questionnaire: "meal assistance service (meal delivery, elderly dining table, etc.)," bath assistance service (help bathing) "and" door-to-door housework ". If one of the services needs to be taken as 1 , none need to be taken as 0	2914	0	1	31.2%	0.463
		Medical Treatment	Select the answers of "door-to-door medical treatment" and "medical escort and escort" in the questionnaire. If one of the services needs to be taken as 1 , none is required, it is taken as 0	2914	0	1	52.0%	0.500
Explained Variable	Type of Care Service Demand	Health Education	Select the answers of the "health education service" service items in the questionnaire. If there is a need for such services, the value is 1, if there is no need, the value is 0	2915	0	1	37.9%	0.485
		Rehabilitation Nursing	Select the answers of "rehabilitation nursing" service items in the questionnaire. If there is a need for such services, the value is 1, if there is no need, the value is 0	2915	0	1	26.3%	0.440
		Psychological Comfort	Select the answers of "psychological counseling / chat and boredom" and "social work services (services carried out by social workers)" in the questionnaire. If one of the services needs to be taken as 1 , none is required, the value is 0	2912	0	1	43.8%	0.496
		Social Support	Select the answer of "respite service (temporarily looking after your family for a period of time)" in the questionnaire. If such service is required, the value is 1, if not required, the value is 0	2916	0	1	20.3%	0.402
